# How does the different pre-hospital healthcare workers dispatch ambulances in the pre-hospital environment in Denmark

**DOI:** 10.1186/1757-7241-22-S2-A8

**Published:** 2015-07-16

**Authors:** Lasse P Bentsen

**Affiliations:** 1 University of Southern Denmark, Odense, Denmark; 2 Falck Danmark A/S, Odense, Denmark

## Background

In Denmark, all ambulance transports are dispatched through the Acute Medical Coordination Centre (AMK). Even though this unit is the only way to request an ambulance, it is experienced by many Emergency Technicians EMTs that the visitation based on patient illness and injury differs widely. The visitation is performed either by an AMK visitation officer (AMK-VO), or a doctor that takes contact to AMK to get the ambulance dispatched. Correct dispatching of emergency services can be a complex affair because AMK cannot see the patients themselves.

## Methods

Three different cases were presented for EMTs, paramedics, and visitation officers at AMK. Each was asked to assign what kind of dispatch level (A, B, or C, where A is the most urgent) they would assign to the patients current need. They were also asked what kind of treatment they think the patient would need from the ambulance and the need for a medical emergency care unit (MECU)

## Results

We had 116 responses (43 assistant EMTs, 36 treating EMTs, 27 paramedics, and 10 AMK-VO's). There was no statistical difference in any of the cases regarding if the respondents would dispatch differently, p = 0.4, 0.2 and 0.4, respectively. AMK-VO's were more likely to anticipate the use of pharmacological intervention in case 2 (p < 0.001) and the use of isotonic NaCl infusion in cases 2 and 3 (p < 0.001) than other respondents.

## Conclusion

Even though the results do not show statistically significant differences, a larger and more structured survey is needed, especially considering the relatively small number of respondents in the AMK-VO group. It should also be noted that the cases were presented in written form, and that visitation normally is performed via phone contact. This could make a difference in how people would dispatch ambulances in reality. The anticipated treatment differed between the groups, when considering isotonic NaCl infusion and pharmacological intervention.

